# Stem Cells of Dental Origin: Current Research Trends and Key Milestones towards Clinical Application

**DOI:** 10.1155/2016/4209891

**Published:** 2016-10-13

**Authors:** Athina Bakopoulou, Imad About

**Affiliations:** ^1^Department of Fixed Prosthesis and Implant Prosthodontics, School of Dentistry, Aristotle University of Thessaloniki, Thessaloniki, Greece; ^2^Aix-Marseille University, CNRS, Institute of Movement Science (ISM), Marseille, France

## Abstract

Dental Mesenchymal Stem Cells (MSCs), including Dental Pulp Stem Cells (DPSCs), Stem Cells from Human Exfoliated Deciduous teeth (SHED), and Stem Cells From Apical Papilla (SCAP), have been extensively studied using highly sophisticated* in vitro* and* in vivo* systems, yielding substantially improved understanding of their intriguing biological properties. Their capacity to reconstitute various dental and nondental tissues and the inherent angiogenic, neurogenic, and immunomodulatory properties of their secretome have been a subject of meticulous and costly research by various groups over the past decade. Key milestone achievements have exemplified their clinical utility in Regenerative Dentistry, as surrogate therapeutic modules for conventional biomaterial-based approaches, offering regeneration of damaged oral tissues instead of simply “filling the gaps.” Thus, the essential next step to validate these immense advances is the implementation of well-designed clinical trials paving the way for exploiting these fascinating research achievements for patient well-being: the ultimate aim of this ground breaking technology. This review paper presents a concise overview of the major biological properties of the human dental MSCs, critical for the translational pathway “from bench to clinic.”

## 1. Introduction

A disparate variety of multipotent postnatal or Adult Stem Cells (ASCs) has been identified over the last decade within the oral cavity, raising the intriguing prospect of several alternative therapies in the burgeoning field of Regenerative Dentistry. Oral ASCs can be classified into dental stem cells, encompassing Dental Pulp Stem Cells (DPSCs) [1], Stem Cells from Human Exfoliated Deciduous teeth (SHED) [2], and Stem Cells From Apical Papilla (SCAP) [3, 4], as well as nondental oral SCs, including Dental Follicle Stem Cells (DFSCs) [5], Periodontal Ligament Stem Cells (PDLSCs) [6], Gingival Mesenchymal Stem Cells (GMSCs) [7], Oral Mucosa Stem Cells (OMSCs) found in the lamina propria of adult human gingiva [8], Bone Marrow Mesenchymal Stem Cells (BMMSCs) from orofacial bones [9], Periosteum-Derived Stem Cells (PSCs) [10], and Salivary Gland-Derived Stem Cells (SGSCs) [11]. All these cells are considered as resident in “stem cell niches” of the respective mesenchymal oral tissues and are referred to as mesenchymal stem cells or multipotent mesenchymal stromal cells (MSCs) [12]. In addition to cells derived from healthy tissues, MSCs can also be isolated from damaged oral tissues, such as inflamed pulp [13, 14] or periapical cysts [15].

There is substantial evidence suggesting that dental MSCs reside in a quiescent, slow-cycling state in the perivascular niches of human pulp or apical papilla [16]. It has been further shown by means of genetic lineage tracing in rodent incisors that MSCs residing in the dental pulp may be of dual origin, consisting of not only NG2+ pericyte cells, whose presence is closely dependent on tissue vascularity, but also MSCs of nonpericyte origin, contributing to tissue growth and repair [17]. Dental MSCs are thought to originate from the cranial neural crest, expressing both MSC and neuroectodermal SC markers. These cells comply with the minimal criteria stipulated by the International Society of Cellular Therapy (ISCT) in 2006 [18], including (1) ability to adhere rapidly to plastic culture surfaces, (2) potential for trilineage differentiation towards osteogenic, adipogenic, and chondrogenic phenotypes under the appropriate inductive conditions, and (3) expression of common MSC markers, such as CD105, CD73, and CD90, in conjunction with lack of expression of CD45, CD34, CD14, CD11b, CD79a, CD19, and HLA-DR. Additionally, dental MSCs are characterized by significant population heterogeneity [19], most probably connected to different stages of developmental commitment, reinforced by epigenetic modifications occurring during their* ex vivo* expansion [20, 21]. Importantly, recent studies have shown the pivotal role of not only stem/progenitor cells but also nonprogenitor supportive cells, such as injured fibroblasts occurring via secretion of multiple growth factors and complement bioactive fragments in dentin/pulp regeneration processes, revealing the significance of all different cellular components of the heterogeneous population [22–25].

Among the important advantages of dental MSCs compared to other SC sources, such as bone marrow and adipose tissues, are their higher proliferative capacity, facilitating* ex vivo* expansion in sufficient cell numbers [26, 27]; easy isolation by noninvasive routine clinical procedures (e.g., extraction of impacted third molars or premolars for orthodontic reasons); and the absence, as reported so far, of major adverse reactions, concerning, for example, teratoma formation following* in vivo* application [28]. Previous studies have shown that DPSCs have the ability to produce single-cell derived Colony Forming Units (CFUs), survive for longer periods without undergoing senescence, and exhibit higher (80–100 times) proliferation rates than BMMSCs [1].

The vast majority of published studies provides evidence on the* in vitro* multilineage differentiation potential of dental MSCs towards osteo/odontogenic, adipogenic, chondrogenic, neurogenic, angiogenic, and myogenic lineages when grown under defined culture conditions [19, 28].* In vivo* studies, mostly in ectopic but less often in orthotopic animal models, have supported their potential to reconstitute functional dentin/pulp complexes when mixed with ceramic substrates (such as, Hydroxyapatite Or Tricalcium Phosphate) [29, 30], as well as other tissues, such as bone [31], cementum [32], blood vessels [33–35], and neural tissues [36, 37]. Most recently, attention has been focused on the biological properties of the plethora of soluble trophic and immunomodulatory cytokines produced by dental MSCs (MSC secretome) because of their angiogenic, neurogenic, and tissue repair properties [38]. Additionally, a growing number of preclinical and few clinical “proof-of-concept” studies that have been initiated provide substantial evidence that dental MSCs and/or their secretome can be successfully utilized for dental [39, 40] and nondental biomedical applications [41].

Taking all the above into consideration, this review paper aims to provide a concise overview of the major biological properties of the adult dental MSCs (including DPSCs, SHED, and SCAP) which are critical for Tissue Engineering (TE) applications; among these properties being of major interest to the dental community is their inherent potential to regenerate highly vascularized (angiogenesis) and innervated (neurogenesis) soft and hard dental tissues (dentin/pulp complex, alveolar bone). Current research trends and key milestone achievements that exemplify their clinical utility in Regenerative Dentistry will be also highlighted.

## 2. Localization and Immunophenotypic Characterization of Dental MSCs

Dental MSCs abundantly express (>95% of the cell population) MSC markers, such as, CD90, CD73, and several Cell Adhesion Molecules (CAMs), mainly integrins but also cadherins [42], with the former being responsible for mediating SC adhesion to Extracellular Matrix (ECM) proteins and the latter for cell-cell interactions [43]. Among these, CD29/b1-integrin, CD49 (subunits b/a_2_-integrin, d/a_4_-integrin, e/a_5_-integrin, and f/a_6_-integrin), CD51/a_v_-integrin, CD61/b_3_-integrin, and CD166/ALCAM have been found to be variably expressed in different types of dental MSCs, including DPSCs, SHED, and SCAP, further indicating the heterogeneity of these cell populations [19, 28, 42]. Other MSC markers, such as CD146, CD105, CD106, and STRO-1, may show variable expression, dependent on the type and maturity of the dental MSC population and on interindividual variations among various cell donors [44]. In particular, STRO-1, a marker that recognizes a trypsin insensitive epitope on perivascular cells [45], has been used in isolating MSCs populations from human dental pulp [46] and apical papilla [47] with enhanced “stemness” properties and osteo/odontogenic differentiation potential. Immunolocalization studies have, in addition, demonstrated that a subpopulation of SCs coexpressing STRO-1, the perivascular marker CD146 [48] and the pericyte antigen 3G5, reside in this niche within the adult pulp [16]. Cells expressing another group of markers (STRO-1, CD90, CD105, and CD146) were also identified together with the vascular and nerve fibers of the pulp tissue [13]. Most recently, [49] it was shown that ALDH1-, CD90-, and STRO-1-positive cells are located in both perivascular areas and nerve fibers of dental pulps, indicating the possibility of the existence of more than one SC niche. Finally, a recent report [50] identified a rare (1.5% by flow cytometry) subpopulation of SCAP, coexpressing NOTCH-3, STRO-1, and CD146, which, according to* in situ* immunostaining, were associated with blood vessels.

All types of dental MSCs also abundantly express nestin (neural stem cells), while the positive presence of other neural crest SC markers (musashi-1, p75, snail-1, -2, slug, Sox-9, etc.) has been also reported and linked to their embryonic origin [51, 52]. Sakai et al. [53] have also shown that the majority of DPSCs and SHED expressed several neural lineage markers, including nestin, Doublecortin (DCX; neuronal progenitor cells), *β*III-tubulin (early neuronal cells), NeuN (mature neurons), GFAP (neural stem cells and astrocytes), S-100 (Schwann cells), A2B5, and CNPase (oligodendrocyte progenitor cells). Other, less commonly found markers, such as CD44, CD9, CD10, CD13, CD59, and MSCA-1, have also been reported as expressed in DPSCs [54], while CD44 and CD13 are also expressed in SHED [55]. Dental MSCs, including DPSCs [56], SHED [57], and SCAP [58], also show variable but increased expression of embryonic SC markers, such as Nanog, Oct3/4, SSEAs (-1, -3, -4, and -5), and to a less extent TRA-1-60 and TRA-1-81 [56], as compared to other MSC types [19], such as BMMSCs. Other pluripotency markers, such as SOX-2 and MYC, not normally found in other ASCs, have been reported in tooth germ-derived MSCs [59]. Finally, dental MSCs lack expression of CD45, CD31, HLA-DR, and, in most studies, CD14, while most but not all [60–62] studies have reported absence of expression of CD117 (c-kit) and CD34. Although the ISCT minimal criteria suggest that the absence of CD34 expression is a prerequisite for defining MSCs, more recent studies indicate that CD34 may be expressed in primitive pluripotent stromal stem cells but is progressively eliminated during cell culturing [63]. It has been previously shown that CD34/c-Kit and STRO-1 coexpression confirm a neural crest-derived DPSC niche [56], while, in more recent studies [61] two different (STRO-1+/c-Kit+/CD34− and STRO-1+/c-Kit+/CD34+) DPSC subpopulations with noticeable differences in their stem cell characteristics have been characterized.

Finally, in a recent study [64], the importance of CD271/NGFR in defining a subpopulation of DPSCs with enhanced odontogenic differentiation potential, as compared to other (CD51/CD140a and STRO-1/CD146) subpopulations also showing odontogenic differentiation capacity, has been emphasized. This is in accordance with studies on BMMSCs showing that CD271/NGFR defines an infrequent but very primitive subset (<1%) of the cell population showing enhanced stem cell characteristics [65].

The immunophenotypic characteristics of dental MSCs are summarized in Table 1.

## 3. Differentiation Potential and Paracrine Activity of Dental MSCs* In Vitro* and* In Vivo*

### 3.1. Osteo/Odontogenic Differentiation Potential of Dental MSCs and Regeneration of Dentin/Pulp- and Bone-Like Tissues

One of the most salient characteristics of dental MSCs concerning dental TE applications rests on their odontogenic differentiation potential. Previous studies have shown that dental MSCs, including DPSCs, SHED, and SCAP, have the capacity to differentiate into odontoblastic lineages* in vitro* and of regenerating dentin/pulp-like complexes or bone-like tissues ectopically and around teeth and implants [29, 31, 66] (the literature summarized in Table 2).

Specifically,* DPSCs* have demonstrated the capacity to differentiate into odontoblastic-like cells with characteristic cell polarity [67]. When seeded onto dentin, DPSCs may convert into odontoblast-like cells with polarized cell bodies and cellular processes extending into the dentinal tubules [68]. In addition, in recently published work using transcriptome analysis of odontoblasts at different stages of maturity, the p38/MAPK signaling has been identified as the crucial pathway to controlling odontoblast secretory activity and therefore a key molecular target for the therapeutic application of DPSCs [40].

Early reports showed that DPSCs mixed with Hydroxyapatite/Tricalcium Phosphate (HA/TCP) led to the formation of ectopic pulp-dentin-like tissue complexes in immunocompromised mice [1, 6, 69]. Iohara et al. [70] combined 3-dimensional cell pellets and Bone Morphogenetic Protein 2 (BMP-2) to induce reparative dentin formation in a dog amputated pulp model. The same group also detailed the possibility of using a subfraction of CD31−/CD146− and CD105+ cells for pulp regeneration [71, 72] and in later studies described the effects of Granulocyte-Colony Stimulating Factor (G-CSF) and host age on pulp regeneration [73, 74]. In another study, DPSCs seeded onto collagen scaffolds in presence of Dentin Matrix Protein 1 (DMP-1) induced the formation of a pulpal-like tissue [75]. Similarly, when implanted in enlarged root canals of immunocompromised mice, DPSCs showed the ability to synthesize newly formed dentin and vascularized pulp-like tissue [76], thus providing prospects for utilization of DPSC transplantation for dentin-pulp regeneration.

Other* in vivo* studies have shown the capacity of DPSCs in bone regeneration in a variety of animal models, including repair of critical-size calvarial defects [77–79] and segmental alveolar defects in a New Zealand rabbit model [80], as well as the capacity for enhancement of implant osteointegration in sites of experimental canine mandibular bone defects [81]. Swine Dental Pulp Stem Cells seeded on TCP scaffolds were also able to regenerate mandibular bone defects in the symphyseal regions of a minipig model [82].

Notably, various scaffolding materials with differing chemical, physical, and mechanical characteristics have been selected for use in pulp/dentin and bone regeneration protocols using dental MSCs, including long-lasting porous bioceramics (e.g., HA, *β*-TCP, or bioactive glasses), natural molecules of medium duration (e.g., collagen, chitosan, hyaluronic acid-based hydrogels, and silk fibroin), and short-life polymers, such as Polyglycolic Acid (PGA), Polylactic Acid (PLA), or their combinations [39, 83]. In addition, injectable hydrogels (including self-assembling multidomain peptides [84] and a commercial blend Puramatrix™) [85] have been suggested for pulp regeneration in the light of their ability to form nanofibrous matrices under physiological conditions. Recent studies have also proposed demineralized/chemically Treated Dentin Matrices (TDMs) [86] or Cryopreserved Treated Dentine Matrices (CTDM) [87], as ideal biologic scaffolds, because of their combination of favorable mechanical properties and ability to act as a reservoir of dentinogenesis-related growth/morphogenetic factors [88]; this is also validated by* in vivo* studies [89, 90]. Finally, strategies to improve stem cell/scaffold interfaces also include incorporation of various bioactive molecules [29], as the third component of the TE triad (cells/scaffolds/growth factors). The application of such growth factors without stem cells, in a cell homing versus cell transplantation strategy, has also been suggested as a more clinically translational approach for dentin-pulp regeneration. Based on this concept, ectopic regeneration of dental pulp-like tissues using basic Fibroblast Growth Factor (b-FGF), Vascular Endothelial Growth Factor (VEGF), or Platelet-Derived Growth Factor (PDGF) with a basal set of Nerve Growth Factor (NGF) and Bone Morphogenetic Protein 7 (BMP-7) has been reported [91], while other researches achieved complete pulp regeneration in pulpectomized mature dog teeth by using a stromal cell-derived factor-1a- (SDF-1a-) loaded silk fibroin scaffold without DPSC transplantation [92].

Significant similarities, but also differences in osteo/odontogenic differentiation potential, have been reported for* SHED*. Pivotal studies by Miura et al. [2] showed that SHED are characterized by osteoinductive capacity* in vivo*, but only a quarter of the clones showed potential to generate ectopic dentin-like tissue. SHED were also able to form an osteoinductive template in immunocompromised mice, inducing the recruitment of host murine osteogenic cells to repair critically sized calvarial defects [93]. Recently, it was shown that both DPSCs and SHED combined with Platelet-Rich Plasma (PRP) were able to regenerate vascularized bone tissue around dental implants in dog and puppy models, respectively [55]. Recent reports have also shown that 5-year cryopreserved SHED were still able to proliferate and undergo osteogenesis without immune reaction in a 9 mm mandibular defect in dogs [94] and to enhance mandibular distraction osteogenesis in a rabbit model [95].

Despite those studies showing the preferential osteogenic versus odontogenic differentiation potential of SHED, other studies also report that SHED are capable of differentiating into functional odontoblasts* in vitro* [2] and of regenerating a tissue with architecture and cellularity resembling the physiologic dental pulp when seeded in biodegradable scaffolds prepared within human tooth slices and transplanted into immunodeficient mice [89]. It has been recently shown that SHED can generate functional dental pulp when injected with scaffolds (Puramatrix or rhCollagen) into full-length root canals [85].

A very recent and interesting study mapping potential molecular differences between SHED and DPSCs identified several differentially regulated genes [96]. Among these high-mobility group AT-hook 2 (HMGA-2) protein, a stem cell-associated marker, together with several proliferation-related genes showed a robust expression in SHED, while ECM genes, such as collagen I, fibronectin, and signaling molecules, such as VEGF, Fibroblast Growth Factor Receptor 1 (FGFR-1), and Insulin Growth Factor Receptor 1 (IGFR-1) were upregulated in DPSCs, suggesting that SHED are more competent in self-renewal and proliferation and DPSCs in signaling and matrix synthesis.

Finally,* SCAP* appear as a cell population similar to, but significantly different from, DPSCs [97]. Although the apical papilla is the precursor tissue of the radicular pulp [18], it is an anatomically distinct area separated by a cell-rich zone. SCAP have been reported to display a higher proliferation rate, number of population doublings, dental tissue regeneration capacity, and STRO-1 expression in comparison with DPSCs [68]. In addition, SCAP have shown a higher expression of survivin and telomerase, two proteins critical for cell proliferation [4]. In contrast, SCAP have been shown to express lower levels of markers, such as Dentin Sialoprotein (DSP), Matrix Extracellular Phosphoglycoprotein (MEPE), transforming growth factor receptor II (TGFbRII), and Vascular Endothelial Growth Factor receptor I (VEGFR1) compared to DPSCs [19]. A recent study demonstrated significant variations in the mineral composition of mineralized tissues produced* in vitro* by various types of dental MSCs [98]. SCAP and SHED produced a more highly mineralized matrix in comparison with DPSCs but with lower crystallinity and carbonate substitution.

Studies have indicated that SCAP are capable of differentiating into odontoblastic-like cells [97] and osteogenic cells [99]* in vitro* and into vascularized dentin/pulp-like complexes, after transplantation into immunodeficient mice, in an appropriate carrier substrate [4, 68]. Additionally, transplantation of SCAP inside a root-shaped HA/TCP block coated with PDLSCs into the extraction socket of a minipig lower incisor demonstrated the successful regeneration of the root/periodontal structure over which a porcelain crown has been placed [100]. Furthermore, SCAP could generate cement/woven bone-like tissue with embedded cementocytes/bone-like cells; however, the precise nature of the mineralized tissue produced was not identified [101].

Although SCAP have not been so closely investigated as DPSCs, several later reports provide significant insight into the particular molecular mechanisms responsible for SCAP biological responses to various microenvironments, providing data pivotally useful for the design of future regenerative strategies for targeted dental TE. Among key inductive factors demonstrated to exhibit a major role in SCAP osteo/odontogenic differentiation are BMP-2 [102], BMP-9 [103], and the conjunction of BMP-2 and VEGF [104]. Other studies have highlighted the importance of Nuclear Factor I-C (NFIC) known to be involved in the regulation of root development [105] and its regulatory interaction with transforming growth factor-*β*1 (TGF-*β*1) in inducing odontogenic transformation of SCAP [106]. In a recent study, Plasminogen activator Inhibitor 1 (PAI-1), has been suggested as pivotal factor in inducing odontogenic differentiation of SCAP [107]. Finally, a number of studies have also closely studied the signaling pathways regulating odontogenic differentiation of SCAP; among these, differential activation of ALK5/Smad2 and MEK/ERK [108], canonical Wnt synergistic with BMP-9 [109], Notch [110], canonical NF-*κ*B [111], and ERK and JNK signaling pathways in combination with a mechanical stress stimulus have been indicated as having a paramount role in the committed differentiation of SCAP [112].

It must be noted that a major problem concerning* in vivo* studies aiming at regenerating functional dentin-pulp complexes or bone around teeth and implants is the fact that the majority have been conducted in ectopic implantation models, mostly subcutaneously into immunocompromised mice [13, 29, 67, 76], and to a less extent in renal capsules of rats [113]. In contrast, only few attempts in orthotropic large-animal models (dogs or mini pigs) have been performed by a sole research group [70–73], probably in view of the considerable economic costs involved together with the ethical issues associated with animal welfare. Most recently, a root implant model in minipigs involving the middle sections of roots from freshly extracted swine incisors filled with scaffolds containing DPSCs and then implanted into the fresh postextraction sockets has been designed. This provided a valuable animal (although not really orthotopic) model simulating clinical situations [114].

Current pulp regeneration protocols have also been recently systematically reviewed by Fawzy El-Sayed et al. [30]. From 1364 screened articles the authors selected five studies for the quantitative analysis complying to specific inclusion/exclusion criteria. They revealed that stem cell transplantation was linked with significantly greater regenerated pulp and dentin per root canal total area when compared with controls. A solitary study reported on capillaries/nerves per unit surface area and found that the density of both nerves and capillaries was noticeably greater in stem/progenitor cell-transplanted pulps compared with controls [72]. The authors emphasized the paucity of quantitative evaluations of the amount of regenerated tissue and the lack of consensus about defining the primary outcomes of the regenerative procedures, including neural, vascular, soft, or hard tissue/dentinal regeneration as the primary limitation of the majority of* in vivo* studies. It was also mentioned that conclusions were drawn on the basis of histological assessments without additional functional innervation and vascularization tests to provide a more comprehensive assessment of functional pulp/dentin regeneration. Interestingly, the majority of studies showed a high risk of selection, performance, detection, and reporting bias. The main causes of this bias were attributed to the fact that none of the studies had performed sample size calculations to enhance statistical power, while lack of standardization of the animal models and type of experimental defects was a cause of significant heterogeneity. In addition, no split-mouth designs were applied, while clustering of statistical units within the same animal was a common practice. Finally, randomization of treatments and blinding of examiners were reported in very few studies.

### 3.2. Angiogenic Properties of Dental MSCs

#### 3.2.1. Endothelial Transdifferentiation Potential of Dental MSCs

Encouraged by the exceptional “plasticity” of dental MSCs, a limited number of studies have attempted to investigate the endothelial transdifferentiation potential of DPSCs [51, 62, 115–118], SHED [119, 120], and SCAP [58, 121] in the presence of specialized angiogenesis-inductive media (summarized in Table 2). The endothelial shift of MSCs in these studies is mainly indicated by the upregulation of typical endothelial cell (EC) markers, such as PECAM-1, VEGFR-2, vWF, and VE-cadherin and further evidenced by functional assays, such as ability to form capillary-like structures on Matrigel or other matrices or by uptake of Acetylated-Low Density LipoProtein Lipase (Ac-LPL), but also by various* in vivo* assays, including mouse Matrigel assays and Chicken Chorioallantoic Membrane (CAM) assays [33, 34, 122].

According to the* in vitro* studies, coculture of DPSCs [123] or SCAP [124] with ECs significantly improved the angiogenic potential of ECs, especially under hypoxic conditions [124, 125]. SHED differentiation into VEGFR-2/CD31 positive EC-like cells has been shown through a VEGF/MEK-1/ERK signaling pathway [120]. Moreover, a VEGFR-2-dependent function of murine DPSCs as pericyte-like cells has been substantiated, since a shRNA knockdown of VEGFR-2 produced a decreased expression of VEGFA, VEGF receptors, and Ephrin B-2 and reduced vascular density of Matrigel plugs* in vivo* [118]. Finally, short-term exposure of SCAP to serum, glucose, and oxygen deprivation (SGOD) conditions has been shown to be potent in eliciting a proangiogenesis program, as evidenced by activation of VEGF/VEGFR and Angiopoietins/Tie pathways [58]. These results confirm that dental MSCs can actually show considerable adaptability to severely adverse microenvironmental conditions, by undergoing a rapid endothelial shift rather than activating apoptosis.

Despite encouraging data, most of the above-mentioned studies actually indicate but fail to prove a functional and homogenous* in vitro* differentiation of MSCs into ECs, suggesting that it might be inaccurate to designate EC-switched MSCs as mature ECs, but rather as an intermediate EC-like population, primarily supporting typical functions of mature ECs or mainly acting in a paracrine way (as analyzed below). Thus, identification of additional microenvironmental cues as well a more detailed understanding of the molecular mechanisms responsible for skewing dental MSCs into mature ECs could constitute a critical step for utilizing them as neoangiogenesis sources in TE.

In addition to* in vitro* data, additional evidence from* in vivo* studies could show that SHED differentiate into ECs when seeded in biodegradable scaffolds and transplanted into immunodeficient mice [89]. DPSCs alone or primarily in coculture with Human Umbilical Vein Endothelial Cells (HUVEC) when encapsulated in three-dimensional peptide hydrogel matrices (PuraMatrix) were able to support cell survival, migration, and capillary network formation and to regenerate vascularized pulp-like tissue after transplantation in mice [126]. Iohara et al. [127] were able to isolate and characterize a highly vasculogenic subfraction of side population (SP) of CD31−/CD146− porcine tooth germ-derived dental MSCs, while in later study the CD31− pulp fraction was used successfully to reconstitute blood flow and capillary density in a mouse hindlimb ischemia model, to induce neurogenesis in a cerebral ischemia model, and finally to reinstitute a vascularized pulp in an ectopic root transplantation model [116]. Similar results were reported for human DPSCs, which showed ability to induce angiogenesis and reduce infarct size in a myocardial infarction rat model [128].

#### 3.2.2. Angiogenic Properties of Dental MSC Secretome

Despite encouraging data on the endothelial transdifferentiation potential of dental MSCs, significant lines of evidence indicate that the rate of MSC engraftment after local or systemic delivery* in vivo* remains problematically low at <10% [129]. This contrasts with several other lines of evidence suggesting that the angiogenic effects of MSCs are primarily derived from secretion of several soluble factors, such as growth factors, cytokines, chemokines, Extracellular Matrix proteins and proteases, or even genetic material [130] as a response to various microenvironmental cues (summarized in Table 1), rather than their endothelial transdifferentiation. There is growing interest in the investigation of MSC “secretome” with the increasing recognition of the paracrine/autocrine role of MSCs to many biological functions, including cell proliferation, differentiation, signaling, apoptosis, angiogenesis, and neurogenesis. Furthermore, the use of cell-free approaches offers several advantages with respect to concerns related to immunogenicity, tumorigenicity, and transmission of infections, which, although currently considered very low for autologous therapies with adult MSCs, are still under investigation in “proof-of-concept” clinical studies being underway in various fields of Medicine and Dentistry.

Dental MSCs (DPSCs and SCAP) have been shown by recent studies to secrete, under various stress conditions, several pro- and antiangiogenetic factors able to stimulate endothelial motility and function [58, 121]. In particular, it has been shown that DPSCs secrete several proangiogenic factors (VEGF, Monocyte Chemotactic Protein 1- (MCP-1), IL-8, Insulin-Like Growth Factor Binding Protein 3 (IGFBP-3), and Urokinase Plasminogen Activator (uPA)) and antiangiogenic factors (Tissue Inhibitor Of Metalloproteinase 1 (TIMP-1), Plasminogen Activator Inhibitor-1 (PAI-1), endostatin, and Thrombospondin-1 (TSP-1)), under serum deprivation conditions [117], while in a later study by the same group differential angiogenic secretome expression was observed among various dental MSC types, including DPSCs, SCAP, and DFSCs [121]. Interestingly, DPSCs and SCAP elicited a predominant proangiogenic effect* in vitro* and* in vivo* compared to DFSCs, which renders them an attractive cell source for angiogenesis applications. Subsequently, it has been shown that, under serum, glucose, and oxygen deprivation (SGOD) conditions, SCAP release higher numbers and amounts of proangiogenic factors (Angiogenin, IGFBP-3, and VEGF) and lower amounts of antiangiogenic factors (Serpin-E1, TIMP-1, and TSP-1) in comparison with SOD or SD alone, providing insights into the optimal preconditioning strategies for SC-based treatment of damaged/ischemic tissues [58]. Most recently, SCAP secretome has been extensively profiled [131]; it was found that a total of 2,046 proteins are released, including chemokines, angiogenic, immunomodulatory, antiapoptotic, and neuroprotective factors, and ECM proteins. SCAP secreted significantly larger amounts of chemokines and neurotrophins than BMMSCs, whereas BMMSCs secreted more ECM proteins and proangiogenic factors.

It is significant to note that secretion of various soluble factors by MSCs may occur either via exocytosis or via release of extracellular vesicles (EVs). These include either exosomes (30–100 nm in size, originating from intracellular microvesicles) or microvesicles (100–1000 nm in size, originating from the plasma membranes) [132]. A recent study showed that DPSC-derived exosomes suppress carrageenan-induced acute inflammation in mice [133]. This was among other reasons attributed to the fact that SHED exosomes contain annexin A1 that acts as mediator of the antimigratory effects of glucocorticoids, thereby suppressing edema formation.

### 3.3. Neurogenic Properties of Dental MSCs

#### 3.3.1. Neurogenic Transdifferentiation Potential of Dental MSCs

Numerous studies so far have highlighted the inherent neurogenic differentiation potential of dental MSCs (summarized in Table 2), attributed to their neural crest embryonic origin [134].* DPSCs* [51, 135–141],* SHED* [36, 57, 142, 143], and* SCAP* [4, 47, 125, 144–146] have shown enhanced potential for differentiation into a variety of neural lineages, including functionally active dopaminergic cells and glial cells, leading proposals for dental MSCs to be used for regenerative therapy of several neurodegenerative diseases [37]. Notably, dental MSCs, while still in an undifferentiated state, constitutively express markers of neural stem/progenitor, as well as mature neural cells, including SOX-2, tenascin C, ENO-2, MAP2ab, c-FOS, Nestin, Neurofilament (NEF-H and NEF-L), Glial Fibrillary Acidic Protein (GFAP), bIII-tubulin, Microtubule-Associated Protein 2 (MAP-2), and many others [143]. However, the data regarding the neural differentiation potential of dental MSCs seem to vary for different cell types and their subpopulations in the vast body of studies published to date [37], preventing safe comparative conclusions regarding the superiority of any one cell type in regenerating neural tissues.

An overview of existing literature actually reveals the wide range of diversity encountered in the neural differentiation protocols used so far by different research groups. This complexity is connected to (1) the culture microenvironment, (2) the application of either single- or in most recent studies multiple-stage differentiation protocols often alternating cell suspension (in the form of spheroids/neurospheres) with adherent cell culture systems, and (3) the biological endpoints explored by each study.

Regarding the cell culture conditions, a variety of substrates, predominantly poly-l-lysine [36, 57, 140], poly-l-ornithine with/without lamin [147], gelatin [4, 47], and more rarely other substrates, have been used, while in most studies direct culture in culture-treated polystyrene [61, 125, 135, 137, 138, 141, 144] forms the commonest practice. However, the absence of comparative studies makes conclusions about the superiority of one substrate over the other impossible. Regarding the neuroinductive culture media, most studies use either the Neurobasal A or conventional primarily Dulbecco's Modified Eagle's Medium (DMEM)/F12 media in their neural differentiation protocols. These are used in conjunction with various neural supplements (most commonly the B27 [36, 125, 142, 144, 148, 149], but also the N2 consisting of a mixture of insulin, transferrin, progesterone, selenium, and putrescine [137] and the insulin-transferrin-selenium (ITS) supplement [54] or their combinations [143]) in a serum-free approach. Alternatively, in other studies, the media are supplemented with conventional fetal calf (FCS) or Fetal Bovine Serum (FBS) at least for the first-stage preincubation phase [135]. In addition to these supplements, various growth factors, mainly Epidermal Growth Factor (EGF) and basic Fibroblast Growth Factor (FGF-2) and, to a less extent, Nerve Growth Factor (NGF), Neurotrophin 3 (NT-3), Brain-Derived Neurotrophic Factor (BDNF), Sonic Hedgehog (SHH), Glial Cell Line-Derived Neurotrophic Factor (GDNF), and so forth, have been used to induce neural maturation. These are additionally supported by neuroinductive small molecules, such as beta-mercaptoethanol, 5-azacytidine, retinoic acid, dibutyryl cyclic adenosine monophosphate (dbcAMP), 3-Isobutyl-1-Methylxanthine (IBMX), Dimethyl Sulfoxide (DMSO), Butylated Hydroxyanisole (BHA), forskolin, and hydrocortisone [37]. All these factors and supplements have been variously used in a number of studies, overall making it impossible to define an ideal culture microenvironment or neural induction approach.

Neural differentiation in the majority of these studies is evaluated by the expression of neural markers (such as NCAM, GFAP, GAP-43, GABA, NeuN, bIII-tubulin, synapsin, NSE, and NFL [37]), while very few have carried out functional assessments. Methods most applied to confirm functional neural transformation include the patch-clamp analysis of the voltage-dependent Na^+^ or K^+^ channels [139–141, 147] and the fluorescent detection of intracellular Ca^2+^ flux upon stimulation with neurotransmitters [135].

Finally, a small number of studies have performed* in vivo* transplantation of dental MSCs to assess cell engraftment and neural marker expression [140] but also for neural disease treatment in various experimental animal models. Predifferentiated SHED-derived neurospheres were applied into the striatum of parkinsonian rats and significant improvement in behavioral impairment as compared to the implantation of control undifferentiated SHED was reported [36]. Similar results were achieved after inducing neural maturation of SHED into dopaminergic neuron-like cells and transplantation in parkinsonian rats [150]. Moreover, transplantation of neural-induced SHED in a rat Spinal Cord Injury (SCI) model led to complete recovery of hindlimb motor function [148]. All of the above studies support that neural preinduction of undifferentiated MSCs before* in vivo* transplantation increases the expression of neural surface receptors and therefore the grafting efficiency into the nervous system, potentially improving clinical outcomes. In a very interesting recent study, the entire apical papilla was transplanted in a SCI (hemisection) model, in comparison to transplantation of human SCAP inside fibrin hydrogels [146]. Significantly, the delivery of SCAP in their original niche (entire apical papilla) improved gait and reduced glial reactivity, as compared to the classical TE approach of cell expansion and delivery in 3D scaffolds. This highlights the importance of the 3D organization of stem cells and the surrounding microenvironment. Finally, another important set of* in vivo* studies were carried out by Sasaki et al. [151, 152]. They used silicone tube conduits filled with a collagen gel containing rat DPSCs and managed to bridge an experimental gap in the rat facial nerve. In a subsequent study, [152] the same group replaced the nonabsorbable silicon material with a degradable PLGA tube that was readily resorbed simultaneously promoting nerve regeneration.

#### 3.3.2. Neurogenic Properties of Dental MSC Secretome

There is a growing body of evidence questioning the ability of dental MSCs to differentiate into functional neurons after transplantation* in vivo* and supports the idea that their neurogenic action is primarily exerted as in the case of angiogenesis through multiple neurotrophic factors found in their secretion products and acting in a paracrine manner (Table 1). Sakai et al. [53] showed that transplantation of DPSCs into rat SCI lesions lead to functional recovery despite only glial rather than neuronal differentiation being observed under these extreme conditions, suggesting a paracrine-mediated action. Mead et al. [153] contended that DPSCs have limited potential to differentiate into neurons and fail to integrate into the retina, after transplantation. The same group found that DPSCs have a more favorable neurotrophic secretome, rich in NGF, BDNF, and NT-3, in comparison with BMMSCs [154], which is efficient in promoting survival and neuritogenesis/axogenesis of bIII-tubulin positive retinal cells after transplantation into the vitreous body of the eye; this effect was neutralized after the addition of specific Fc-receptor inhibitors, overall suggesting a paracrine effect. Various other studies have reported on the existence of multiple neurotrophic factors, including NGF, BDNF, NT-3, CNTF, GDNF, VEGF, and FGF-2 [53, 154–156] in DPSC secretome. Finally, DPSCs mobilized by G-CSF were shown to secrete a panel of neurotrophic and angiogenic factors (BDNF, GDNF, IGF, NGF, and VEGF) capable of regenerating myelinated fibers in a rat sciatic nerve defect model [157].

A series of studies on the neuroregenerative/neuroprotective properties of SHED secretome have been also published by the group of Mita et al. using various experimental neural disease models. They have found that SHED-derived, serum-free Conditioned Medium (SHED-CM) improved cognitive function in an Alzheimer's disease mouse model [158] and enhanced recovery of focal cerebral ischemia in rats after intranasal administration [159]. Additionally, SHED-CM after intracerebral administration in mice with perinatal hypoxia-ischemia-induced brain injury generated an anti-inflammatory environment, reduced tissue loss, and significantly improved the neurological outcome by converting a M1 proinflammatory to an M2 anti-inflammatory environment. The latter was primarily attributed to the combined secretion of MCP-1 and the Secreted Ectodomain of Sialic Acid-Binding Ig-Like Lectin-9 (ED-Siglec-9) among 28 proteins detected in SHED-CM [160]. These results have been also validated by other groups that used SHED-CM for peripheral nerve regeneration across nerve gaps on rat sciatic nerve gap models [161]. A recent study also investigated the neuroprotective role of SHED-derived exosomes, highlighting another mechanism of their paracrine-mediated action [162].

In contrast to DPSCs and SHED, little data exist so far on the neurogenic activity of SCAP secretome. A recent study [145] demonstrated that SCAP release BDNF responsible for triggering directed axonal targeting both* in vitro* and* in vivo*, as shown by microfluidic and Matrigel implant experiments. Yu et al. also detected several neurotrophic factors in SCAP secretome, including Midkine (MDK), Pleiotrophin (PTN), Mesencephalic Astrocyte-Derived Neurotrophic Factor (MANF), Neuroblast Differentiation-Associated Protein (AHNAK), and Neurophilin 2 (NRP2).

Thus, we seem to be able to conclude that the neuroregenerative/neuroprotective properties of dental MSCs are primarily exerted through a paracrine mechanism rather than on their potential for* in vivo* differentiation into mature neural phenotypes. Current research trends are focusing on the preconditioning strategies to enhance neurogenic properties of dental MSC secretome, as an effective surrogate therapeutic module for stem cell transplantation therapies in the treatment of neurodegenerative diseases.

## 4. Establishment of Clinical-Grade Dental MSCs and Challenges to Be Overcome before Clinical Application

Despite the very promising results of the plethora of TE approaches published to date on the application of dental MSCs for the regeneration of various tissues, very few clinical trials mainly in the form of new methodological paradigms or “proof-of-concept” (phase I/II, safety/efficacy) studies have been conducted or are currently being conducted. This is in complete contrast to the rapidly growing number of clinical trials using other MSC sources (mainly BM-MSCs) in treatment of various bone/articular, cardiovascular, neurological, immune, and blood pathologies (data found on https://clinicaltrials.gov/). The unique biological value of MSCs lies in the combination of differentiation potential into tissue-forming cells and the paracrine-mediated revascularization/reinnervation of the regenerated tissues, under an immunosuppressive/immunoregulatory “deck” limiting probability for adverse reactions [163].

However, one of the basic factors still hindering extensive clinical application of MSC-based therapies is among others the difficulty encountered in the* ex vivo* expansion of clinical-grade, xeno-free MSCs under Good Manufacturing Practice (GMP) conditions, as described in the EU Regulation 2003/94/EC [164] (*GMP is that part of quality assurance which ensures that products are consistently produced and controlled to the quality standards appropriate to their intended use and as required by the marketing authorization*) and in compliance to the EU regulations (1394/2007) [165] established for the clinical use of Advanced Therapy Medicinal Products (ATMPs). These have been defined as “*biological medicinal products containing or consisting of living cells or sub-cellular fractions with biological functions*.” AMTPs do not belong to the same category of drugs or transplants because (1) they contain viable allogeneic or autologous cells undergoing* ex vivo* substantial manipulations (as defined in the EU Regulation 1394/2007, Annex 1) and (2) they may be applied in “non-homologous use,” that is, at sites not physiologically present or to perform biological functions they do not usually participate in. ATMPs are considered Cell-Based Medicinal Products (CBMPs) when containing living cells or tissues. CBMPs are “*medicinal products presented as having properties for, or used in or administered to, human beings with a view to treating, preventing or diagnosing a disease in which the pharmacological, immunological or metabolic actions are carried out by cells or tissues*” [166].

The recent literature on the subject has questioned whether epigenetic (e.g., homing receptor/ligand expression, cytokine/growth factor production, lineage commitment/differentiation, and programmed senescence) [20, 21] and genetic alterations (e.g., transformation, fusion, and gene transfer) occurring during expansion culture [167] may affect the therapeutic potential of stem cells in a positive or negative way. For example, the changes shown might be beneficial for site-specific application depending on the target tissues but adverse for systemic administration or vice versa. Since development of adequate numbers of high quality SCs at early passages is a prerequisite for any safe cell therapy treatment, considerable effort has been put into evaluating the consequences of the cultivation process on stem cell behavior, in particular, in developing reliable standardization protocols in the form of Standard Operating Procedures (SOPs) to be routinely applied to characterize (1) phenotypic and genetic stability of cultured dental MSCs, (2) efficacy in regenerating target tissues, (3) the permitted population doubling before senescence becomes a problem, (4) the absence of microbial, viral, fungal, mycoplasma, endotoxin, or other contamination in cultured cells, and (5) lack of tumorigenicity, toxicity, and immunogenicity, something highlighted in recent reports discussing current challenges towards clinical application of dental MSCs [168, 169]. It becomes clear from these reports that the lack of reliable characterization methods and reference standards for the evaluation of each of the above mentioned important parameters presents a major hurdle for the development of cGMP-grade cells and respective CBMPs.

Among other parameters, significant efforts have been made to replace animal sera used in conventional media due to their highly variable and often unknown composition, the immunological risks associated with serum proteins, and the potential of transmission of prion diseases [170]. Considering the significant impact of serum components in MSC maintenance and multilineage differentiation [171], efforts to replace it with autologous or allogeneic sera or with proprietary serum-free media of unknown formulations by different companies have yet to be validated for their efficacy, while their use is still restricted by the prohibitive cost. The need for development of chemically defined media which can maintain “stemness” without adversely affecting MSC function, immunoregulatory properties, and phenotype remains a significant problem to be overcome for cGMP production of MSCs [172].

Apart from establishment of clinical-grade dental MSCs, SOPs must be also developed for each of the successive steps leading to clinical application including (1) scaling-up of culture systems to produce the desired cell numbers based on the targeted therapeutic goal (upstream process); this might range from thousands to billions of cells depending on the size of the defect; a major problem to achieve this lies on the significant variability in donors and the derived cell lines, which may significantly complicate the culture scale up for high-throughput production in automated and parallel culturing systems [173]; (2) harvesting (preferably by mechanical means or by a cGMP enzymatic process using recombinant enzymes and avoiding porcine-derived trypsin or similar reagents [174]), volume reduction, and isolation of the desired cell populations (downstream possess); in particular, for cell isolation, molecular-tagging based methods have been employed to purify dental MSCs by using specific molecular markers; among these methods, fluorescence-activated cell sorting (FACS) has been mostly used offering the advantage of multiparametric analysis for several markers [175]; although FACS systems have been recently upgraded to cGMP function [176], they have limited capacity for large-scale MSC processing and significantly high costs; the same reservations can be made for the magnetism and adsorption-based cell separation systems (MACS), which are considered to represent the “gold” standard method for cell purification, but they also have limited scalability and low efficacy to obtain high cell numbers [177]; (3) loading into appropriate carriers and preserving the final ATMP in safe conditions for immediate or later application. The latter requires robust cryopreservation processes with minimal adverse effects on cell survival and “stemness” characteristics [178]. While the conventional slow-freezing and rapid-thawing method in liquid nitrogen or its vapor phase is the “state-of-the-art” method [179], other methods such as vitrification by the “open pulled straw” method using high cryoprotectant concentrations and ROCK inhibitor treatment together with flash freezing in liquid nitrogen have been proposed to result in higher cell survival rates [180]. However, direct contact with liquid nitrogen is considered a major drawback, as it may increase the risk of cross-contamination among samples. It still remains quite challenging that all of the above-mentioned steps, which are routinely used for research purposes, have to be optimized, upgraded, and standardized to be carried out under cGMP conditions and followed by quality assessments to secure safety and efficacy of the delivered cell-based products, making the whole process quite complicated and extremely time-consuming.

Other scientific, technological, policy, and commercial development challenges and hurdles have also to be addressed before extensive clinical application of dental MSC therapies using commercially available ATPMs to replace the biomaterial-based treatment modalities currently being used in clinical dentistry, in a solid, evidence-based manner. Another challenging point to be considered before application of dental MSC therapies in clinical dentistry is that most currently applied biomaterials and clinical methodologies have despite reported biological and technical complications high overall survival and success rates [181]. In addition, they are related to nonlife threatening diseases; therefore any novel alternative therapies need to be shown to have marked superiority to be established as clinically routine processes.

In contrast to medical literature, very limited published work exists so far on the development of clinical-grade dental MSCs and related ATMPs. In an effort to avoid serum-containing media, Tarle et al. [182] evaluated the capacity of chemically defined serum-free culture systems to effectively expand and maintain the stem cell properties of SHED and PDLSCs. Although these cells proliferated at lower rates in serum-free conditions, their multilineage differentiation potential and differential expression of 84 stem cell-associated genes showed only minor differences compared to the serum-containing medium, thus validating application of such serum-free, cGMP conditions for their safe and effective expansion. The same group proposed use of fibronectin an important serum component for optimizing the initial recovery of DPSCs from pulp biopsies under serum-free conditions [183]. Lizier et al. [184] developed a protocol of scaling-up large numbers of dental MSCs at early passages by mechanical transfer (i.e., without enzymatic treatment) into new culture dishes, thus minimizing risk of loss of their “stemness.” Other novel cell culture systems for large-scale expansion such as cell factories and bioreactors have been proposed as extremely effective for other oral MSC types [169]. However, no studies exist so far on the application of these systems to dental MSC expansion, which would be important towards optimizing 3D microenvironments for targeted dental TE.

A recent report [185] described manufacturing strategies of DPSC-based ATMPs to improve safety, efficacy, and consistency of their GMP production. The authors proposed the use of impacted third molars of young healthy donors between 5 and 7 Nolla's developmental stage (i.e., from complete crown upto one third of root completed) as ideal dental MSC sources. Regarding culture conditions, they proposed explant culture instead of enzymatic dissociation, although both methods have been associated with advantages and disadvantages [186, 187], both being capable of recovering approximately 1 million cells from one third molar within 2 weeks. The authors also proposed the precoating of culture plates with a mixture of human placental collagens I and III, use of GMP reagents, such as TrypLe® or Accutase®, and serum-free, clinical-grade culture media. Finally, they considered typical MSC markers such as CD105, CD90, and CD73 proposed by ISCT as being expressed by several MSC populations and therefore being nonspecific and proposed a large and multiparametric immunophenotyping as crucially important.

**Figure 1:**
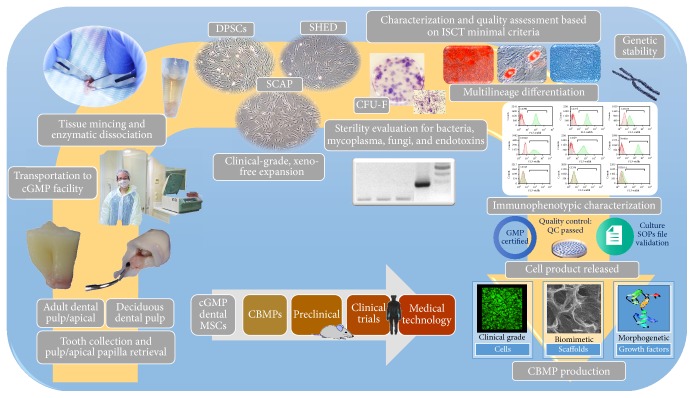
Process for the development of clinical-grade, xeno-free, GMP-compliant dental MSC cultures and of the respective dental MSC-based CBPMs for preclinical and clinical evaluation.

Based on the above, the process for the development of clinical-grade, xeno-free, GMP-compliant dental MSCs cultures and of the respective dental MSC-based CBPMs for preclinical and clinical evaluation is illustrated in Figure 1.

## 5. Dental MSCs-Based Clinical Trials

A significant number of studies have already been published using MSCs for the regeneration of orofacial bones, including sinus augmentation and regeneration of large- (cleft palate, alveolar ridge augmentation, maxillary replacement, mandibular fracture, replacement, and osteoradionecrosis cases) or small-size bone defects. These studies, mainly comprised of case reports/series together with few randomized controlled clinical trials (RCTs), have been systematically reviewed by Padial-Molina et al. [188] and Jakobsen et al. [189]. In the majority of these studies, BMMSCs and to a lesser extent other MSC types such as periosteum-derived MSCs or adipose tissue-derived MSCs have been used. These cells were cultured in growth media containing bovine serum, autologous serum, or other growth media and the cells either were preinduced or were not preinduced towards osteogenic differentiation before cell transplantation.

In contrast, very few clinical studies using dental MSCs have been published so far. Two successive studies by the group of Papaccio et al. [190, 191] reported on the use of autologous DPSCs, combined with a collagen sponge, to repair human mandible bone defects after extraction of third molars. The authors reported optimal vertical bone repair three months after surgery and complete restoration of periodontal tissue back to the second molars. They also evaluated the bone quality three years after transplantation and found that an entirely compact rather than spongy bone was the final outcome, without any serious clinical implications. Notably, these studies were performed in the absence of the above-described universally accepted protocols of GMP-compatible production of DPSCs. Nakashima et al. [192] published a series of studies in the potential of mobilized DPSCs to regenerate pulp in dog pulpectomized teeth and based on this they have initiated a clinical trial with pending announcement. This will provide significant insight into the potential for bringing dental MSC-based pulp regeneration into clinical reality. Finally, besides already published studies, an electronic search in the https://clinicaltrials.gov/ database under the key words “mesenchymal stem cells” resulted in 595 clinical trials (excluding 11 which have been withdrawn), applying MSCs in various medical conditions, while only 4 clinical trials have been initiated using dental stem cells, as analytically described in Table 3.

## 6. Conclusions

Despite the constraints and limitations of current research approaches, it is safe to conclude that dental MSCs, including DPSCs, SHED, and SCAP, have been extensively studied in the past years by the dental research community using highly sophisticated* in vitro* and* in vivo* systems; this has led to a substantial understanding of their unique biological properties. As a result, bioengineering of various constituents of dental tissues such as dentin, pulp, or alveolar bone using dental MSC-based TE approaches has now been achieved. In addition, “proof-of-concept” studies for whole-tooth regeneration [193–195] are among the most fascinating recent advances, however, despite the intriguing possibilities that are opened up, there is still considerable need for further work to attain “clinical reality.” Nevertheless, the major challenge still remains: how can and will the results of this extremely time-consuming, laborious, and costly research be translated into clinical therapeutic modules available to the patient; who is the final recipient of this groundbreaking technology. To consolidate the clinical utility of dental MSCs and/or their secretome in Regenerative Dentistry, there is pressing need for the initiation of well-designed RCTs aiming at the regenerative treatment of various oral tissues. This will allow a full understanding of the potential risks involved in the use of these technologies and spur efforts to surmount any problems and create a viable therapy option, a potential milestone in the application of science to clinical settings.

## Figures and Tables

**Table 1:** Marker expression in dental MSCs (SHED, DPSCs, and SCAP) and factors identified in their secretomes.

Dental MSCs	Positive markers	Negative markers	Factors in secretome involved in angiogenesis	Factors in secretome involved in neurogenesis
Stem cells from Human Exfoliatd Deciduous teeth (SHED)	CD90, CD73, CD105, CD146, STRO-1, CD44, CD13 Nestin, DCX, *β*ΙΙΙ-tubulin, NeuN, GFAP, S-100, A2B5, CNPase Nanog, Oct3/4, SSEAs (-3, -4)	CD11b CD45 CD34 CD14 CD19 CD43	VEGF-A, VEGF-C, EG-VEGF (PK-1), HGF, IGF-1, FGF-2, SDF-1, SCF, EGF, TIMP-1, -2, MMP-2, -3, -9, MCP-1, ANG, TGF-b	BDNF, GDNF, MCP-1, ED-Siglec-9, IL-6, NRCAM, GDF-15, NCAM-1, TACE, Nidogen-1 NRG-1, TIMP-1, -2, HGF, SCF, MMP-2, -3, -9, decorin, IL-22, IL28A, IL-29, osteopontin, SCF, ANG, VEGF-A, EG-VEGF, VEGF-C, growth hormone, insulin, PIGF, TGF-b

Dental Pulp Stem Cells (DPSCs)	CD90, CD73, CD105, CD146, STRO-1, CD106, CD29, CD49, CD51, CD61, CD166, ALDH1, 3G5, CD44, CD9, CD10 CD13, CD59, MSCA-1, CD81, CD24 CD271/NGFR, Nestin, DCX, *β*ΙΙΙ-tubulin, NeuN, GFAP, S-100, A2B5, CNPase, musashi-1, p75, snail-1, -2, slug, Sox-9 Nanog, Oct3/4, SSEAs (-1, -3, -4, -5), Notch-1, -2, -3	CD45 CD34 CD14, CD19 CD31, CD117, CD133 HLA-DR	VEGF, uPA, IL-8, TSP-1, IGFBP-3, TIMP-1, -4, MMP-9, PAI-1 (serpin E1), endostatin, ANGPT-1, ANG, DPPIV, EDN-1, PTX-3, PEDF (serpin F1), PDGF-AA and PDGF-AB/BB, MCP-1	NGF, BDNF, NT-3, CNTF, GDNF, MCP-1, VEGF, FGF-2, PDGF-AA and PDGF-AB/BB, MMP-9, ANG, TIMP-1, -4

Stem Cells from Apical Papilla (SCAP)	CD90, CD73, CD105, CD146, STRO-1, CD106, CD29, CD49, CD51, CD61, CD166, ALDH1, 3G5, CD44, CD9, CD10 CD13, CD59, MSCA-1, CD34, CD81, CD24, c-Kit CD271/NGFR, Nestin, NSE, CNPase, musashi-1, p75, snail-1, -2, slug, Sox-9 Nanog, Oct3/4, SSEAs (-1, -3, -4, -5), TRA-1-60-, TRA-1-81, Notch -2, -3	CD14 CD18 CD34 CD45 CD117 CD150	ANGPT-1, ANG, DPPIV, EDN-1, PTX-3, PEDF (serpin F1), IGFBP-1, -2, -3, TIMP-1, -4, TSP-1, VEGF, uPA, Activin A, HGF, FGF-7, PIGF (serpin E1), TGFb, CXCL-16, persephin, NRG1-b1, MCP-1	MDK, NEGF-1 (PTN), NEGF-2, CXCR4, MANF, AHNAK, NRP2, ANG, TIMP-1, -4, CXCL-16, NRG1-b1, MCP-1

**Table 2:** Key references on the osteo/odontogenic, angiogenic, and neurogenic differentiation potential of dental MSCs *in vitro* and *in vivo.*

Dental MSCs	Osteo/odontogenic differentiation		Angiogenic differentiation		Neurogenic differentiation	
	*In vitro*	*In vitro *and/or* in vivo *(bone or dentin/pulp)	*In vitro*	*In vitro *and/or* in vivo*	*In vitro*	*In vitro *and/or* in vivo*
Stem cells from Human Exfoliated Deciduous teeth (SHED)	[26, 98]	[2, 53, 55, 60, 67, 82, 85, 89, 90, 93–95]	[67]	[119, 120]	[57, 67, 142, 143]	[36, 53, 148, 150]

Dental Pulp Stem Cells (DPSCs)	[14, 21, 26, 49, 51, 54, 59, 61, 88, 97, 123]	[1, 13, 53, 55, 56, 62, 67, 69–81, 84, 113, 114]	[51, 59, 67, 115, 123]	[62, 116–118, 121, 122, 126–128]	[51, 54, 59, 61, 67, 135–139, 143, 144, 147]	[53, 56, 116, 140, 141, 151, 152]

Stem Cells from Apical Papilla (SCAP)	[4, 44, 47, 50, 97, 98, 102, 104–106, 108, 110–112]	[52, 76, 99–101, 103, 106, 109]	[58, 124, 125]	[121]	[47, 52, 144]	[145, 146]

**Table 3:** Clinical trials using dental MSCs currently being registered at https://clinicaltrials.gov/ database.

	Title	Type of cells	Treatment	Disease	Stage	Number of subjects	(Estimated) completion date	Endpoints	Results	Clinicaltials.gov identifier
(1)	Periodontal Regeneration of Chronic Periodontal Disease Patients Receiving Stem Cells Injection Therapy	Allogeneic human DPSCs	Local injection at the local periodontal defects	Chronic periodontal disease	Recruiting	40	Dec. 2016	Improvement of baseline alveolar bone volume and clinical parameters, including probing depth (PD), Clinical Attachment Level (CAL), Quigley-Hein plaque Index (QHI), Bleeding on Probing (BoP)	Not reported yet	NCT02523651

(2)	Use of Mesenchymal Stem Cells for Alveolar Bone Tissue Engineering for Cleft Lip and Palate Patients	SHED (autologous)	Application of MSCs inside a collagen and hydroxyapatite biomaterial (Geistlich Bio-Oss®) into the defect	Alveolar bone TE for cleft lip and palate patients (secondary alveolar graft after completion of orthodontic treatment)	Unknown	5	Mar. 16	Amount and quality of regenerated bones (CT scans)	In all 5 patients bone formation closing the alveolar cleft was observed after 6 months	NCT01932164

(3)	Revitalization of Immature Permanent Teeth with Necrotic Pulps Using SHED Cells	SHED (autologous)	Application of scaffold-free SHED-derived pellet	Immature permanent teeth with pulp necrosis	Recruiting	80	Jul. 2017	Pulp status evaluated by dental pulp vitality tester; pulp revascularization examined by laser Doppler flowmeter; and the index of clinical examination Also, the degree of apical closure; the rate of increase in root length; and the change of root canal wall thickness.	Not reported yet	NCT01814436

(4)	Effect on Allogenic Mesenchymal Stem Cells on Osseointegration of Dental Implants	Allogeneic human DPSCs	The implant is dipped in the stem cell solution for 3 minutes so that the cells adhere to the titanium implant surface before placement at the osteotomy site	Improvement of implant osseointegration	Enrolling by invitation	10	Feb. 2017	Evaluation of primary and secondary stability is measured using Resonance Frequency Analysis (RFA).	Not reported yet	NCT02731586

## Abbreviations

Ac-LPL: Acetylated-Low Density LipoProtein Lipase
AHNAK: Neuroblast Differentiation-Associated Protein
ANG: Angiogenin
ANGPT-1: Angiopoietin 1
ASCs: Adult Stem Cells
ATMPs: Advanced Therapy Medicinal Products
BDNF: Brain-Derived Neurotrophic Factor
BHA: Butylated Hydroxyanisole
BMMSCs: Bone Marrow Mesenchymal Stem Cells
BMP-2: Bone Morphogenetic Protein 2
CAM: Chicken Chorioallantoic Membrane
CAMs: Cell Adhesion Molecules
CBMPs: Cell-Based Medicinal Products
CFUs: Colony Forming Units
CM: Conditioned Medium
CNTF: Ciliary Neurotrophic Factor
CTDM: Cryopreserved Treated Dentine Matrices
CXCL-16: Chemokine (C-X-C motif) Ligand 16
dbcAMP: Dibutyryl Cyclic Adenosine Monophosphate
DCX: Doublin or Lissencephalin-X (encoded by* DCX* gene), most known as Doublecortin
DFSCs: Dental Follicle Stem Cells
DMEM: Dulbecco's Modified Eagle's Medium
DMP-1: Dentin Matrix Protein 1
DMSO: Dimethyl Sulfoxide
DPPIV: Dipeptidyl Peptidase-4
DPSCs: Dental Pulp Stem Cells
DSP: Dentin Sialoprotein
ECM: Extracellular Matrix
EDN-1: Endothelin 1
ED-Siglec-9: Secreted Ectodomain of Sialic Acid-Binding Ig-Like Lectin-9
EGF: Epidermal Growth Factor
EG-VEGF (PK1): Endocrine Gland-Derived Vascular Endothelial Growth Factor or Prokineticin-1
EVs: Extracellular Vesicles
FBS: Fetal Bovine Serum
FCS: Fetal Calf Serum
FGF: Fibroblast Growth Factor
FGFR-1: Fibroblast Growth Factor Receptor 1
GABA: Gamma-Aminobutyric Acid
GAP-43: Growth Associated Protein 43
G-CSF: Granulocyte-Colony Stimulating Factor
GDF-15: Growth Differentiation Factor 15
GDNF: Glial Cell Line-Derived Neurotrophic Factor
GFAP: Glial Fibrillary Acidic Protein
GMP: Good Manufacturing Practice
GMSCs: Gingival Mesenchymal Stem Cells
HA/TCP: Hydroxyapatite/Tricalcium Phosphate
HGF: Hepatocyte Growth Factor
HMGA-2: High-Mobility Group AT-hook 2
HUVEC: Human Umbilical Vein Endothelial Cells
IBMX: 3-Isobutyl-1-Methylxanthine
IGF-1: Insulin-like Growth Factor 1
IGFBP: Insulin-like Growth Factor Binding Protein
IGFR-1: Insulin Growth Factor Receptor 1
IL: Interleukin
ISCT: International Society of Cellular Therapy
MANF: Mesencephalic Astrocyte-derived Neurotrophic Factor
MAP-2: Microtubule-Associated Protein 2
MCP-1: Monocyte Chemotactic Protein 1
MDK: Midkine
MEPE: Matrix Extracellular Phosphoglycoprotein
MMP: Matrix Metalloproteinase
MSCs: Mesenchymal Stem Cells or Mesenchymal Stromal Cells
NCAM-1: Neural Cell Adhesion Molecule 1
NFIC: Nuclear Factor I-C
NFL: Neurofilament
NGF: Nerve Growth Factor
NRCAM: Neuronal Cell Adhesion Molecule
NRG-1-B-1: Neuregulin Beta 1
NRP-2: Neurophilin 2
NSE: Neuron Specific Enolase
NT-3: Neurotrophin 3
OMSCs: Oral Mucosa Stem Cells
PAI-1 (serpin E1): Plasminogen Activator Inhibitor-1
PDGF: Platelet-Derived Growth Factor
PDLSCs: Periodontal Ligament Stem Cells
PEDF (serpin F1): Pigment Epithelium-Derived Factor
PGA: Polyglycolic Acid
PIGF: PhosphatidylInositol-Glycan Biosynthesis Class F
PLA: Polylactic Acid
PRP: Platelet-Rich Plasma
PSCs: Periosteum-Derived Stem Cells
PTN: Pleiotrophin
PTX-3: Pentraxin 3
SCAP: Stem Cells from Apical Papilla
SCF: Stem Cell Factor
SCI: Spinal Cord Injury
SCs: Stem cells
SDF-1a: Stromal Cell-Derived Factor-1a
SGSCs: Salivary Gland-Derived Stem Cells
SHED: Stem Cells from Human Exfoliated Deciduous teeth
SHH: Sonic Hedgehog
SOPs: Standard Operating Procedures
TACE: Tumor Necrosis Factor-A Converting Enzyme
TDMs: Treated Dentin Matrices
TE: Tissue Engineering
TGFb: Transforming Growth Factor Beta
TGFbRII: Transforming Growth Factor Beta Receptor II
TIMP: Tissue Inhibitor of Metalloproteinase
TSP-1: Thrombospondin-1
uPA: Urokinase Plasminogen Activator
VE-cadherin: Vascular Endothelial cadherin
VEGF: Vascular Endothelial Growth Factor
VEGFR-1: Vascular Endothelial Growth Factor Receptor I
vWF: von Willebrand Factor.
